# circHIPK2-mediated σ-1R promotes endoplasmic reticulum stress in human pulmonary fibroblasts exposed to silica

**DOI:** 10.1038/s41419-017-0017-4

**Published:** 2017-12-13

**Authors:** Zhouli Cao, Qingling Xiao, Xiaoniu Dai, Zewei Zhou, Rong Jiang, Yusi Cheng, Xiyue Yang, Huifang Guo, Jing Wang, Zhaoqing Xi, Honghong Yao, Jie Chao

**Affiliations:** 1 Department of Physiology, School of Medicine, Southeast University, Nanjing, Jiangsu 210009 China; 2 Department of Pharmacology, School of Medicine, Southeast University, Nanjing, Jiangsu 210009 China; 3 Key Laboratory of Developmental Genes and Human Disease, Southeast University, Nanjing, 210096 China; 4 Department of Respiration, Zhongda Hospital, School of Medicine, Southeast University, Nanjing, Jiangsu 210009 China; 5 Third Clinical Medical College, Nanjing University of Chinese Medicine, Nanjing, Jiangsu 210023 China

## Abstract

Silicosis is characterized by fibroblast accumulation and excessive deposition of extracellular matrix. Although the roles of SiO_2_-induced chemokines and cytokines released from alveolar macrophages have received significant attention, the direct effects of SiO_2_ on protein production and functional changes in pulmonary fibroblasts have been less extensively studied. Sigma-1 receptor, which has been associated with cell proliferation and migration in the central nervous system, is expressed in the lung, but its role in silicosis remains unknown. To elucidate the role of sigma-1 receptor in fibrosis induced by silica, both the upstream molecular mechanisms and the functional effects on cell proliferation and migration were investigated. Both molecular biological assays and pharmacological techniques, combined with functional experiments, such as migration and proliferation, were applied in human pulmonary fibroblasts from adults to analyze the molecular and functional changes induced by SiO_2_. SiO_2_ induced endoplasmic reticulum stress in association with enhanced expression of sigma-1 receptor. Endoplasmic reticulum stress promoted migration and proliferation of human pulmonary fibroblasts-adult exposed to SiO_2_, inducing the development of silicosis. Inhibition of sigma-1 receptor ameliorated endoplasmic reticulum stress and fibroblast functional changes induced by SiO_2_. circHIPK2 is involved in the regulation of sigma-1 receptor in human pulmonary fibroblasts-adult exposed to SiO_2_. Our study elucidated a link between SiO_2_-induced fibrosis and sigma-1 receptor signaling, thereby providing novel insight into the potential use of sigma-1 receptor/endoplasmic reticulum stress in the development of novel therapeutic strategies for silicosis treatment.

## Introduction

Silicosis is an occupational disease caused by long-term exposure to high levels of dust containing excessive free silica (crystalline silicon dioxide, SiO_2_) in mining and other dusty occupational environments^1,2^. Although preventive efforts have been made for many decades, silicosis remains a potentially fatal, incurable, and disabling pulmonary disease characterized by silicotic nodule formation and pulmonary interstitial fibrosis^3^. Silicosis is especially prevalent in undeveloped countries because of poor surveillance^4^. The incidence and prevalence of silicosis are markedly increasing, and effective therapies are not currently available^5^. Despite the many studies conducted to investigate the toxicity of crystalline silica in the last several decades, to date, the exact mechanism of silicosis remains elusive.

Sigma-1 receptor (σ-1R) is a subtype of the sigma receptor family that is expressed in the endoplasmic reticulum (ER); it has two transmembrane segments and two steroid binding domains, forming a pocket that is the binding site for cholesterol, steroids, sphingolipids^6^, and a wide variety of natural or synthetic ligands from different classes, such as opioids, antipsychotics, psychostimulants, alkaloids, or antidepressants^7^. σ-1R has been associated with many diseases, including cocaine addiction^8^, stroke^9^, Alzheimer’s disease^10^, amnesia^11^, retinal degeneration^12^, and cancer^13–15^. The molecular action of σ-1R was recently revealed to be a ligand-regulated receptor chaperone via ER stress (ERS)^15^. The ER is a specialized perinuclear organelle responsible for synthesis, folding, modification, and delivery of proteins to their target sites^16–18^. Various physiological and pathological conditions may affect ER homeostasis, ultimately causing ERS^19,20^. Although ERS is involved in different types of pulmonary disease^21–23^, σ-1R-associated ERS in pulmonary fibrosis has received little attention.

A recent study suggested that non-coding RNA is involved in σ-1R regulation^24^. Circular RNAs (circRNAs), as a new type of non-coding RNA, have been identified as competing endogenous RNAs that bind miRNAs by complementary base paring^25^. Moreover, circRNAs are also reported to modulate the cell cycle in the formation of complexes with proteins^26^. Recent study suggest that circRNA-homeodomain-interacting protein kinase-2 (circHIPK2) may act as an endogenous miR-506-3p sponge, leading to an increase in σ-1R^27^, whereas its host gene-HIPK2 is involved in cell growth modulation, apoptosis, proliferation and tumor progression^28–31^. Therefore, these studies lend strong support to the hypothesis that circHIPK2, descendent from exon 2 of the HIPK2 gene, may be related in fibroblast activation.

In the current study, σ-1R-associated ERS was upregulated in human pulmonary fibroblasts exposed to SiO_2_, which subsequently induced fibroblast activation. Involvement of circHIPK2 in σ-1R regulation revealed novel functional circRNAs in SiO_2_-induced fibrosis and suggested that the circHIPK2/σ-1R/ERS pathway may be a potential therapeutic target for silicosis.

## Methods

### Reagents

SiO_2_, 80% of which had particle diameters of <5 μm, was purchased from Sigma (S5631), selected via sedimentation according to Stokes’ law, acid-hydrolyzed, and baked overnight (200 °C, 16 h)^32^. The silica samples for the cell experiments were suspended in phosphate-buffered saline (PBS) at a concentration of 5 mg/ml, and the volume of administration was 20 μl/well in 24-well plates at a dosage of 50 µg/cm^2^. Fetal bovine serum (FBS), normal goat serum and Dulbecco’s modified Eagle’s medium (DMEM; #1200-046) were purchased from Life Technologies. GlutaMax Supplement (35050-061) was obtained from Gibco. Lentiviral vectors carrying circHIPK2-siRNA were obtained from HANBIO. Control siRNA (sc-37007) was purchased from Sigma-Aldrich. Antibodies against σ-1R were obtained from Invitrogen. Santa Cruz Biotechnology, Inc. The antibody against α-SMA (SAB5500002) was purchased from Sigma, Inc. The antibody against COL1A2 (BS1530) and COL3A1 (BS1531) were purchased from BioWorld (St. Louis Park, MN, USA).

### Cell culture

Human pulmonary fibroblasts from adults (HPF-a) were purchased from ScienCell and cultured in DMEM supplemented with 10% FBS, 100 U/ml penicillin, 100 μg/ml streptomycin and 2 mM L-GlutaMAX (Gibco) at 37 °C in a humidified 5% CO_2_ atmosphere.

### Lentiviral transduction of HPF-a

P3-4 HPF-a was transfected with LV-GFP lentivirus (HANBIO Inc., Shanghai, China) as previously described^32,33^.

### Immunofluorescence staining

Immunofluorescence staining was performed as previously described^34^.

### Western blotting

Western blotting assays were used to determine the cellular protein levels as previously described^32^. Each western blot assay was repeated at least three times.

### Hoechst 33342 stanining

HPF-a grown on poly-l-lysine-coated glass cover slips were staining using Hoechst 33342(10 μg/ml) after treated with BD1047 for 2 h and SiO_2_ for 24 h. In brief, HPF-a were stained with Hoechst 33342 for 5 min at room temperature and then fixed in 4% PFA for 20 min. The population of apoptosis cells were observed under a fluorescence microscope. Five fields per well were randomly selected for apoptosis cell counting^32^.

### Bromodeoxyuridine (BrdU) labeling

Cells were prepared on poly-l-lysine-coated glass cover slips. 10 μM BrdU (Yeasen, 40204ES60) in Dulbecco’s phosphate-buffered saline was added 4 h before fixation. After fixing in 4% PFA at 4 °C overnight, cells were denatured in 2 N HCl at room temperature for 30 min and then rinsed in 0.1 M borate buffer (pH 8.0, 300 μl/well) for 10 min. After incubation in blocking solution (PBS containing 10% normal goat serum and 0.3% TX-100) for 1 h, cells were then treated with mouse anti-BrdU antibody (1:200; SC-32323, Santa Cruz) overnight at 4 °C. After a wash stage with PBS, cells were incubated with donkey anti-mouse (conjugated to Alexa-Fluor 576) secondary antibodies for 2 h at room temperature. Washed three times in PBS, cells were mounted using mounting solution (Prolong Gold antifade reagent with 4',6-diamidino-2-phenylindole (DAPI); P36931, Life Technologies). The slides were imaged using a fluorescence microscope (Olympus IX70, Olympus America, Inc., Center Valley, PA, USA). Five fields per well were randomly selected for cell counting.

### Cell migration assay

The cell migration capacity in a 2D culture system was evaluated using an *in vitro* scratch assay as previously described^32^.

### Cell viability assessment using the MTT assay

Cell viability was measured using the 3-(4,5-dmethylthiazol-2-yl)-2,5-diphenyltetrazolium bromide (MTT) assay as previously described^32^.

### Real-time PCR

Total RNA was isolated from cells and subjected to reverse transcription using a PrimeScript RT Master Mix Kit (TaKaRa, RR036). Real-time PCR was performed using the following primers: human circHIPK2 (forward primer: 5’-CTGTGTGCTCCACCTACTTG-3’; reverse primer: 5’-TACCCAGTCATGTCCCAGTTG-3’), human GAPDH (forward primer: 5’-ACCATCTTCCAGGAGCGAGAT-3’; and reverse primer: 5’-GGGCAGAGATGATGACCCTTT-3’). Relative quantification was performed using TaKaRa SYBR Premix Taq (TbI RNase H Plus) (TaKaRa, RR420).

### Fluorescence *in situ* hybridization (FISH)

Cellular circHIPK2 expression was detected using FISH with a mixture of biotin-labeled DNA oligo probes that were specific for either endogenous or ectopically expressed circHIPK2. A biotin-labeled scrambled sequence was used as a negative control. In brief, cells were freshly fixed in 4% paraformaldehyde (PFA) for 15 min at room temperature, followed by two washes with PBS, immersion in 70% ethanol overnight at 4 °C, permeabilization with 0.25% Triton X-100 for 15 min, and two 15-min washes with saline-sodium citrate (SSC) buffer. In situ hybridization was performed overnight at 37 °C with 10 pM biotin-labeled DNA oligo probes in hybridization buffer (HB), followed by serial washes with SSC buffer. The samples were incubated in blocking buffer (1% bovine serum albumin and 3% normal goat serum in PBS) for 1 h at room temperature and were then incubated with an anti-biotin HRP antibody (1:200) in blocking buffer overnight at 4 °C. The samples were washed with PBS twice, each for 2 min. DNA was stained with DAPI. Cell images were captured using a fluorescence microscope (Olympus BX53, Olympus America, Inc.).

### Gel contraction assay

Gel contraction assay was performed as previously described^5^.

### Statistical analyses

Statistical analysis was performed using Student’s *t*-test or two-way analysis of variance (ANOVA) with SigmaPlot 11.0. The tests used are indicated in the figure legends. The ANOVA results were considered significant at a *p* < 0.05.

## Results

### Effects of SiO_2_ on σ-1R expression and collagen production in HPF-a

**Fig. 1:**
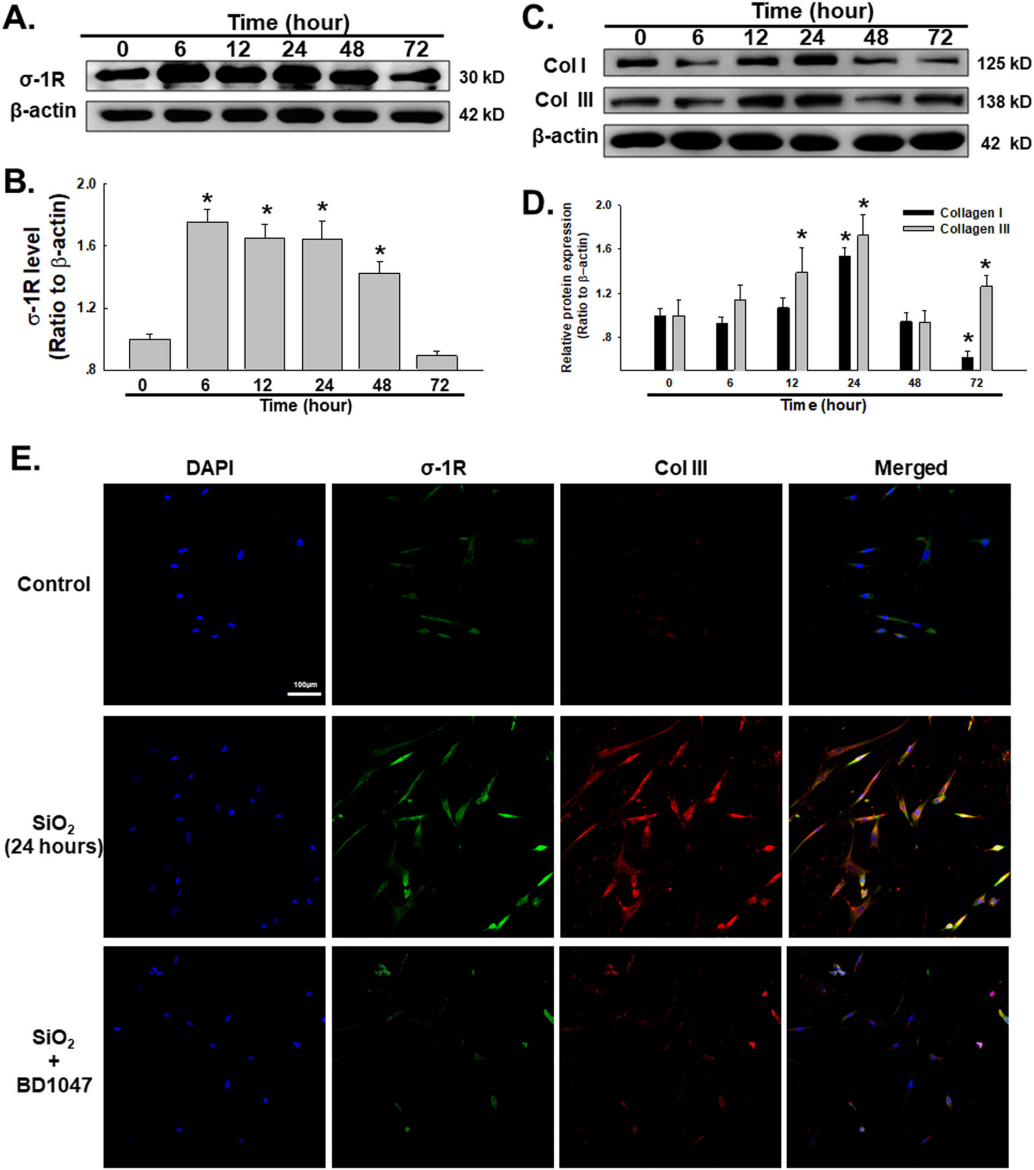
Effects of SiO_2_ on σ-1R expression and collagen production in HPF-a **a** Representative western blot showing SiO_2_-induced σ-1R upregulation in a time-dependent manner. **b** Densitometric analysis of σ-1R from five experiments; * *p* < 0.05 vs the control group. **c** SiO_2_ upregulated collagen I and collagen III expression levels in a time-dependent manner. **d** Densitometric analysis of collagen I and collagen III from five experiments; * *p* < 0.05 vs the control group. **e** Representative immunocytochemical images showing that SiO_2_ increased σ-1R and collagen III expression levels in HPF-a, which was attenuated by pretreatment of BD1047 (10 μM)

Previous reports have indicated that σ-1R is expressed in the lung; however, little is known about the effect of σ-1R on the progression of silicosis. Based on previous dosage experiments, 50 μg/cm^2^ was selected for all the relating experiments^35–39^. To establish whether σ-1R is involved in silicosis, the HPF-a cell line was exposed to SiO_2_ and assessed for σ-1R expression. Immunoblotting results demonstrated that σ-1R was upregulated in the presence of SiO_2_ at 6, 12, 24 and 48 h (Fig. 1a and Fig. 1b). Moreover, collagen I and collagen III production levels were also significantly increased in the presence of SiO_2_, with a peak at 24 h (Fig. 1c and Fig. 1d). Immunocytochemistry assays confirmed the upregulation of σ-1R and collagen III induced by SiO_2_ in HPF-a and the co-localization of σ-1R and collagen III, whereas collagen III induced by SiO2 was attenuated by pretreatment of BD1047, a specific σ-1R inhibitor (Fig. 1e), indicating a role for σ-1R in fibroblast activation induced by SiO_2_.

### Involvement of σ-1R in functional changes induced by SiO_2_ in HPF-a

**Fig. 2:**
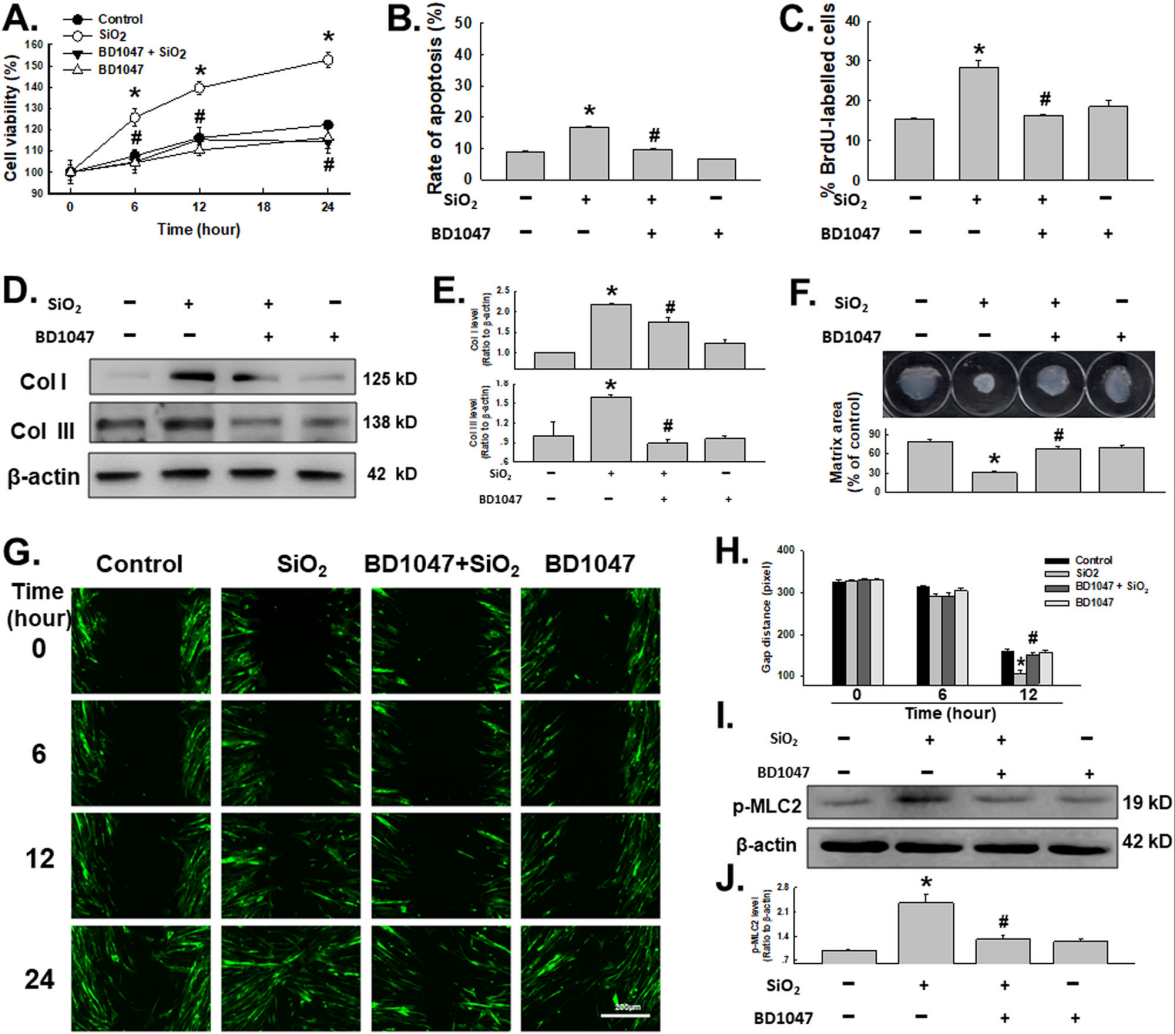
Involvement of σ-1R in functional changes induced by SiO_2_ in HPF-a **a** MTT assay showing that the SiO_2_-induced increase in cell viability was abolished by a 1-h pretreatment with BD1047 (10 μM); *n* = 5; *p <0.05  vs the control group at the corresponding time point. **b** Hoechst 33342 staining demonstrating that apoptosis induced by SiO2 was attenuated by pretreatment of BD1047(10 μM) in HPF-a; *n* = 5; *p < 0.05 vs the control group, ^#^ *p* < 0.05 vs the SiO_2_ group. **c** BrdU labeling assay demonstrating that proliferation induced by SiO_2_ was attenuated by pretreatment of BD1047(10 μM) in HPF-a; *n* = 5; *p < 0.05 vs the control group, ^#^ *p* < 0.05 vs the SiO_2_ group. **d** Representative western blot showing that SiO_2_-induced increased collagen I and III expression levels were attenuated by BD1047. **e** Densitometric analyses of collagen I and collagen III expression from five separate experiments; *p < 0.05 vs the control group; ^#^ *p* < 0.05 vs the SiO_2_ group. Representative immunocytochemical images showing that the σ-1R and collagen III co-localization induced by SiO_2_ was attenuated by pretreatment with BD1047. **f** Representative images and data of gel contraction assay demonstrating that cell activation induced by SiO_2_ was attenuated by pretreatment of BD1047(10 μM) in HPF-a; *n* = 5; *p < 0.05 vs the control group, ^#^ *p* < 0.05 vs the SiO_2_ group. **g** Representative images showing the SiO_2_-induced increase in cell migration using a scratch assay was attenuated by pretreating HPF-a with BD1047. **h** Quantification of the scratch gap distances from six independent experiments; *p < 0.05 vs. the corresponding time point in the control group; ^#^ *p* < 0.05 vs the corresponding time point in the SiO_2_ group. **i** Representative western blot showing that SiO_2_-induced phosphorylation of MLC2 was attenuated by pretreatment with BD1047. **j** Densitometric analysis of the phosphorylation of MLC2 expression from five experiments; *p < 0.05 vs the control group

Pulmonary fibrosis in the late stage of silicosis is characterized by upregulation of fibroblast proliferation and migration^40^. Although σ-1R is involved in cell proliferation and migration in the central nervous system (CNS), the role of σ-1R in the peripheral system remains unclear. To establish the functional relevance of the changes in σ-1R expression, BD1047, a specific σ-1R inhibitor, was applied. As shown in Fig. 2a, SiO_2_ induced a significant increase in HPF-a viability, which was attenuated by pretreatment with BD1047 (10 µM) for 2 h. To further understand the increase of cell viability induced by SiO_2_, both cell apoptosis and proliferation characteristic were analyzed. As shown in Fig. 2b and c, although SiO_2_ induced slight increase in cell apoptosis in HPF-a (<20%), SiO2 increase significant increase in cell proliferation, whereas both cell apoptosis and proliferation induced by SiO2 were attenuated by pretreatment of BD1047. Meanwhile, pretreatment with BD1047 also inhibited collagen I and III induction by SiO_2_ (Fig. 2d, e). Moreover, the matrix contraction assay was conducted, unveiling that pretreatment of BD1047 impaired gel contraction induced by SiO_2_ exposure (Fig. 2f). Next, we explored the roles of σ-1R in SiO_2_-mediated cell migration. As shown in Figs. 2g, h, SiO_2_ induced increased HPF-a migration, which was attenuated by pretreatment with BD1047. To further understand the mechanism of σ-1R in cell migration, myosin light chain 2 (MLC2) levels were measured. Phosphorylation of MLC2 by Rho-associated coiled-coil-forming protein kinase (ROCK), demonstrated to act as an effector downstream of Rho, can increase the contractility of actomyosin, which forms stress fibers and cell protrusions, resulting in cell migration^41,42^. As shown in Fig. 2I, j, MLC2 phosphorylation significantly increased in HPF-a exposed to SiO_2_, which was attenuated by pretreatment with BD1047. MLC2 phosphorylation has been shown to have important roles in promoting actin disassembly and cell detachment in non-muscle cells^43,44^. Excessive myosin activity may destabilize central stress fibers^44,45^, suggesting the role of the Rho-ROCK-MLC2 pathway in cell migration induced by σ-1R.

### SiO_2_ induces ERS in HPF-a

**Fig. 3:**
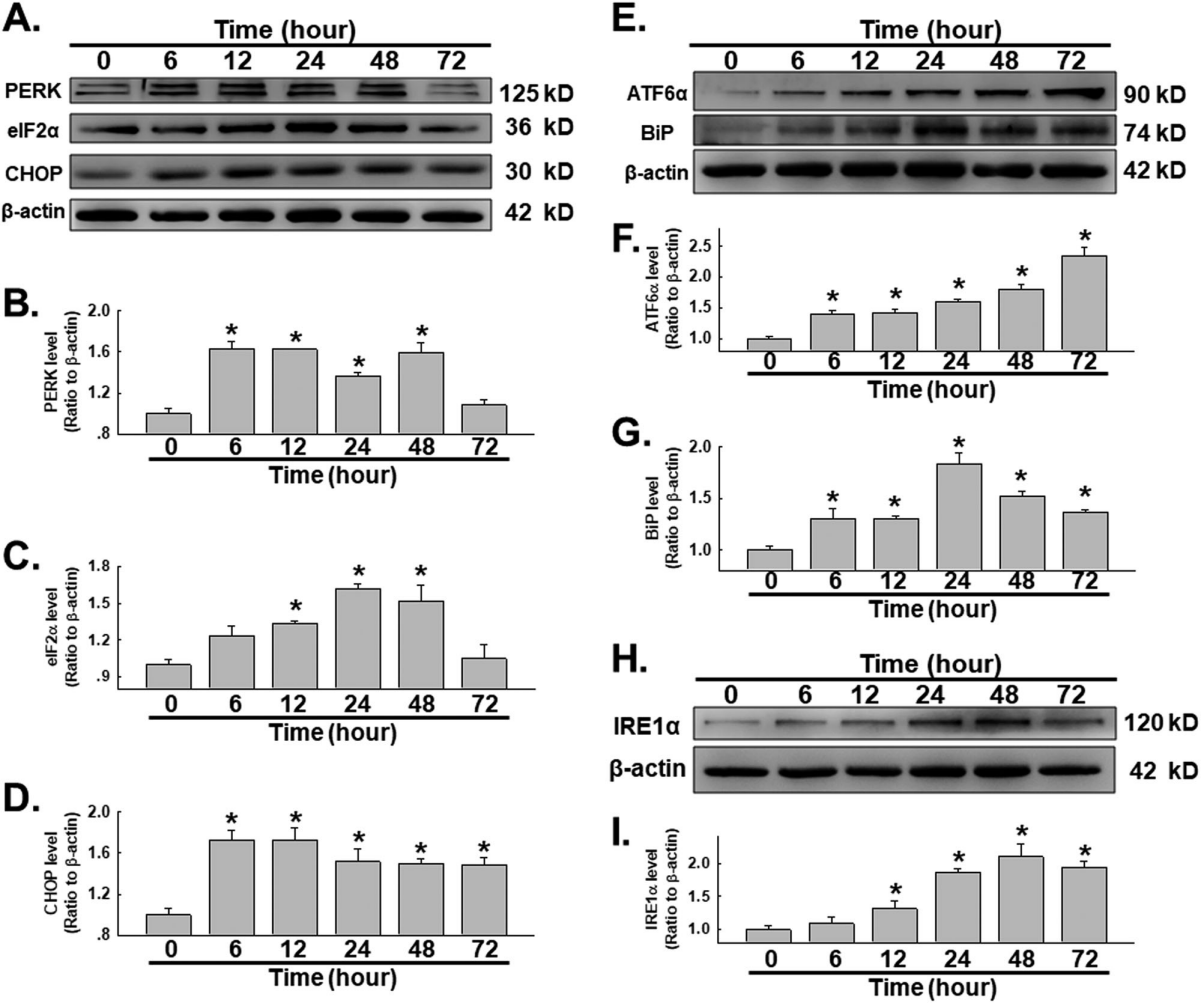
SiO_2_ induces ER stress in HPF-a **a** Representative western blot showing that SiO_2_ induced increased PERK, eIF2α, and CHOP expression. Densitometric analysis of PERK **b**, eIF2α **c** and CHOP **d** protein expression levels from five independent experiments; *p < 0.05 vs the control group. **e** Representative western blot showing that SiO_2_ induced increased ATF6α and BiP expression. Densitometric analysis of ATF6α **f** and BiP **g** protein expression levels from five independent experiments; *p < 0.05 vs the control group. **h** Representative western blot showing that SiO_2_ induced increased IRE1α expression. **i** Densitometric analysis of IRE1α protein expression from five independent experiments; *p < 0.05 vs the control group

σ-1R is a ligand-regulated receptor chaperone triggered via ERS^15^. Whether upregulation of σ-1R also induces functional changes in HPF-a via ERS remains to be clarified. The protein kinase RNA-like ER kinase (PERK)/eukaryotic initiation factor 2α (eIF2α)/C/EBP homologous protein (CHOP) pathway was tested first. SiO_2_ administration induced increases in the expression levels of PERK, eIF2α, CHOP (Fig. 3a–d). Binding immunoglobulin protein (BiP) is a master regulator of the ERS response; thus, we next investigated BiP expression and that of the ERS transcriptional enhancer associated activating transcription factor 6α (ATF6α). HPF-a exposed to SiO_2_ showed increased levels of BiP and elevated ATF6α (Fig. 3e–g). Then, the involvement of the inositol-requiring protein-1α (IRE1α) pathway in SiO_2_-induced ERS was investigated. As shown in Fig. 3h–i, HPF-a treated with SiO_2_ showed upregulation of IRE1α expression in a time-dependent manner. Taken together, these results demonstrate the activation of ERS in HPF-a cells exposed to SiO_2_.

### σ-1R is involved in ERS induced by SiO_2_

**Fig. 4:**
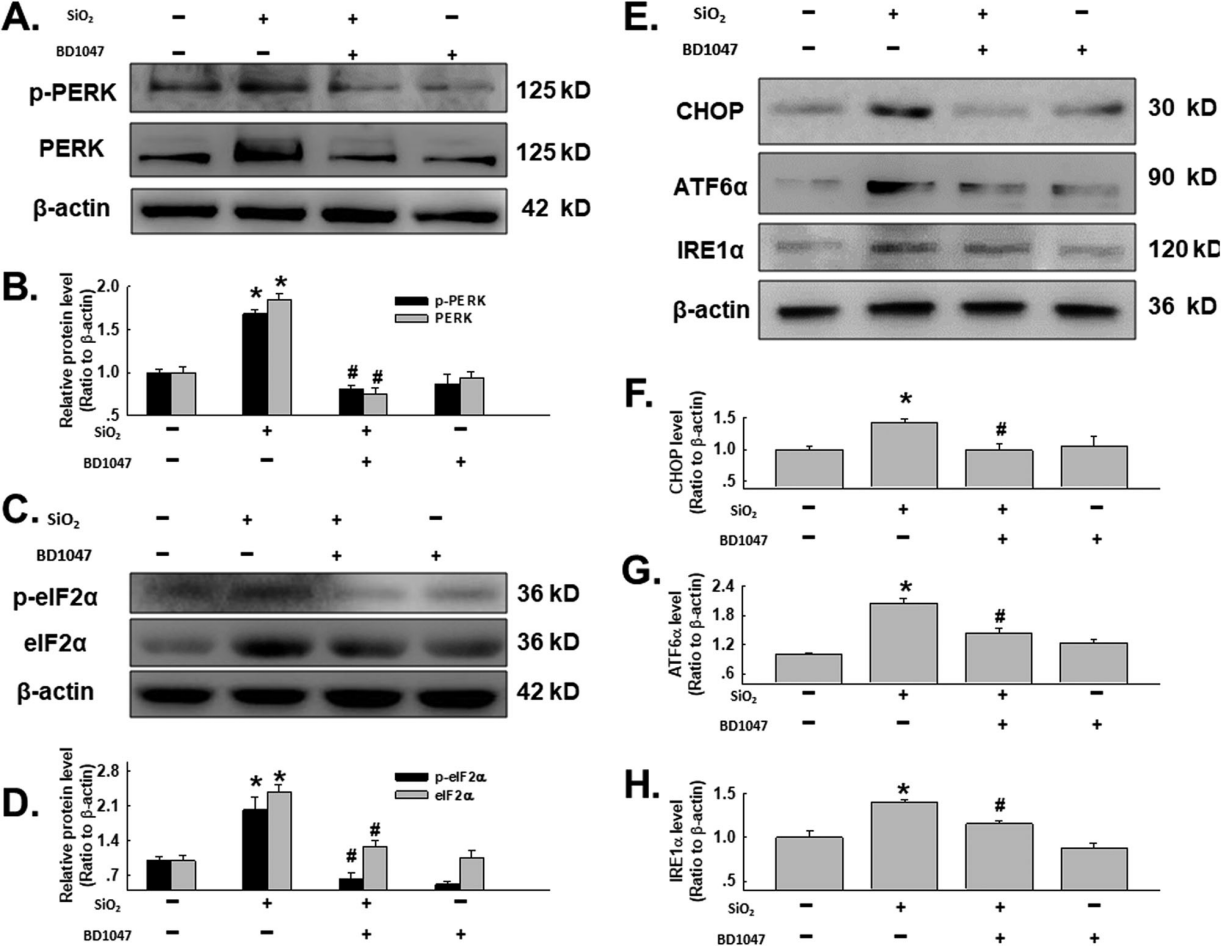
σ-1R is involved in ERS induced by SiO_2_ **a** Representative western blot depicting the effect of BD1047 on PERK phosphorylation. **b** Densitometric analysis of PERK and p-PERK protein expression levels from five independent experiments; *p < 0.05 vs the control group; ^#^ *p* < 0.05 vs the SiO_2_ group. **c** Representative western blot depicting the effect of BD1047 on eIF2α phosphorylation. **d** Densitometric analysis of eIF2α and p-eIF2α protein expression levels from five independent experiments; *p < 0.05 vs the control group; ^#^ *p* < 0.05 vs the SiO_2_ group. **e** Representative western blot depicting the effect of BD1047 on CHOP, ATF6α and IRE1α protein expression. Densitometric analysis of CHOP **f**, ATF6α **g**, and IRE1α **h** protein expression levels from five independent experiments; *p < 0.05 vs the control group; ^#^ *p* < 0.05 vs the SiO_2_ group

To confirm the role of σ-1R in ERS upregulation induced by SiO_2_, BD1047 was applied. As shown in Fig. 4a, b, the exposure of SiO_2_ induced not only upregulation but also phosphorylation of PERK, whereas pretreatment with BD1047 reversed the inductions by SiO_2_. Upregulation of nonphosphorylated and phosphorylated eIF1α induced by SiO_2_ was also attenuated by pretreatment with BD1047 (Fig. 4c–d). Moreover, the induction of CHOP, ATF6α and IRE1α by SiO_2_ was attenuated by pretreatment with BD1047 (Fig. 4e–h), indicating an σ-1R/ERS pathway in fibroblast activation induced by SiO_2_.

### SiO_2_ induced ERS-mediated fibroblast functional changes

**Fig. 5:**
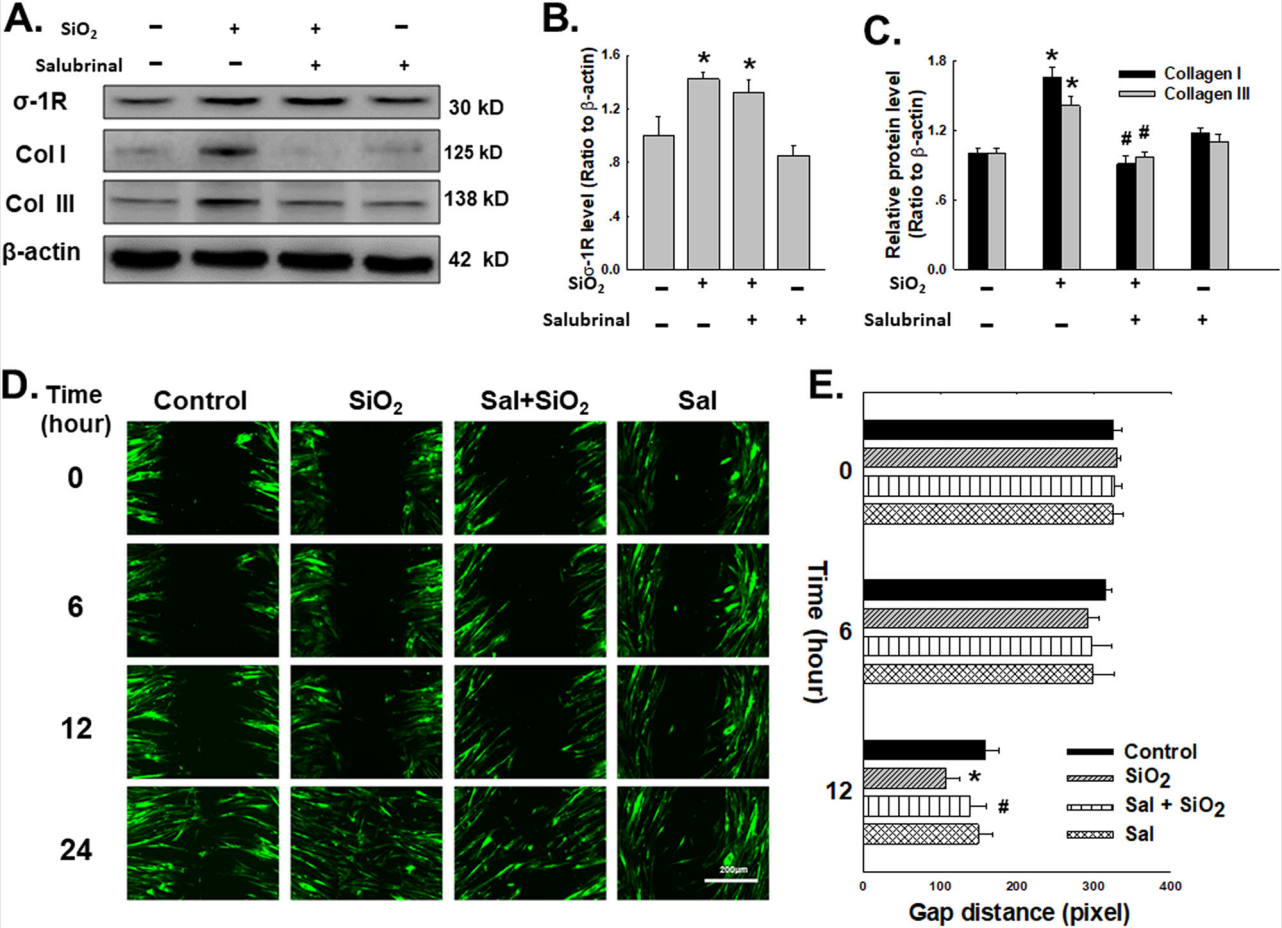
SiO_2_ induced ERS-mediated fibroblast functional changes **a** Representative western blot depicting the effect of salubrinal (10 μM) on σ-1R and collagen I and III expression. Densitometric analyses of σ-1R **b** and collagen I and III **c** expression levels from five separate experiments; *p < 0.05 vs the control group; ^#^ *p* < 0.05 vs the SiO_2_ group. **d** Representative images showing that the SiO_2_-induced increase in cell migration in a scratch assay was attenuated by pretreating HPF-a with salubrinal. **e** Quantification of the scratch gap distances from six independent experiments; *p < 0.05 vs. the corresponding time point in the control group; ^#^ *p* < 0.05 vs the corresponding time point in the SiO_2_ group

After determining that the upregulated ERS associated with σ-1R was induced by SiO_2_, the roles of those changes in fibroblast migration and collagen production were investigated. To this end, salubrinal, a specific inhibitor of ERS, was applied. As shown in Fig. 5a–c, whereas salubrinal did not affect σ-1R induction by SiO_2_, salubrinal attenuated collagen I and III upregulation induced by SiO_2_. In a 2D migration experiment, HPF-a migration was activated by the exposure to SiO_2_, which was then inhibited after blocking the function of ERS with salubrinal. (Fig.5d–e)

### circHIPK2 is involved in regulating σ-1R after SiO_2_ exposure in HPF-a

**Fig. 6:**
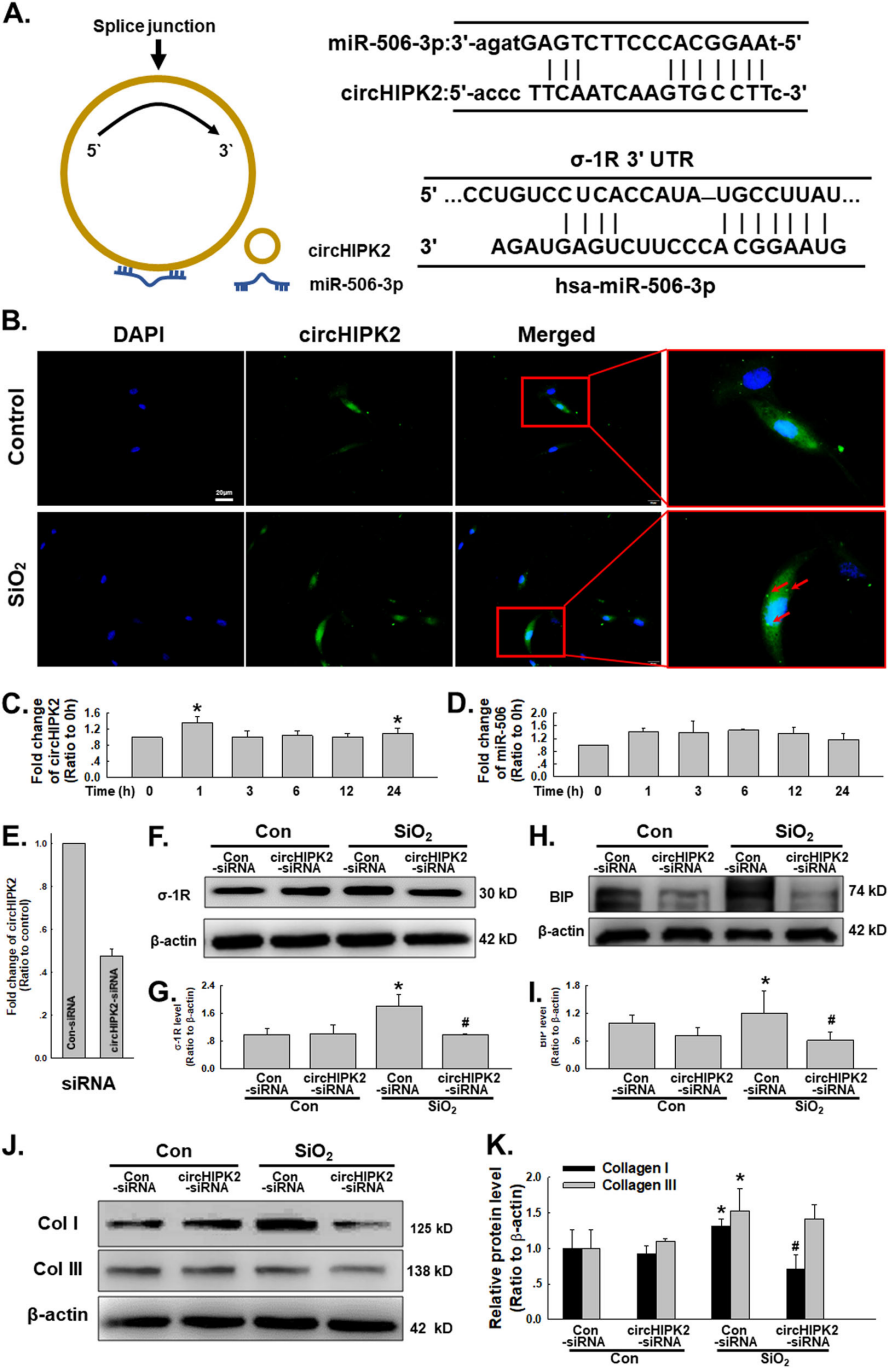
**circHIPK2 is involved in regulating σ-1R after SiO**_**2**_**exposure in HPF-a** **a** Bioinformatics analysis showing that circHIPK2 contains one site complementary to miR-506-3p and two miR-506-3p binding sites in σ-1R. **b** FISH assay showing circHIPK2 expression in HPF-a increased after SiO_2_ exposure; circHIPK2 was labeled with fluorescein isothiocyanate. **c** qRT-PCR assay showing SiO_2_ induced circHIPK2 upregulation from six independent experiments; *p < 0.05 vs the control group. **d** qRT-PCR assay showing SiO_2_ had no effect on miR-506 expression in six independent experiments; *p < 0.05 vs the control group. **e** qRT-PCR assay showing siRNA knockdown of circHIPK2 inhibited circHIPK2 expression in HPF-a; *n* = 5. **f** Representative western blot depicting the effect of siRNA of circHIPK2 on σ-1R expression. **g** Densitometric analyses of σ-1R expression from five separate experiments; *p < 0.05 vs the control group; ^#^ *p* < 0.05 vs the SiO_2_ group. **h** Representative western blot depicting the effect of siRNA of circHIPK2 on BiP expression. **i** Densitometric analyses of BiP expression from five separate experiments; *p < 0.05 vs the control group; ^#^ *p* < 0.05 vs the SiO_2_ group. **j** Representative western blot depicting the effect of siRNA of circHIPK2 on collagen I and III expression. **k** Densitometric analyses of collagen I and III expression from five separate experiments; *p < 0.05 vs the control group; ^#^ *p* < 0.05 vs the SiO_2_ group

**Fig. 7:**
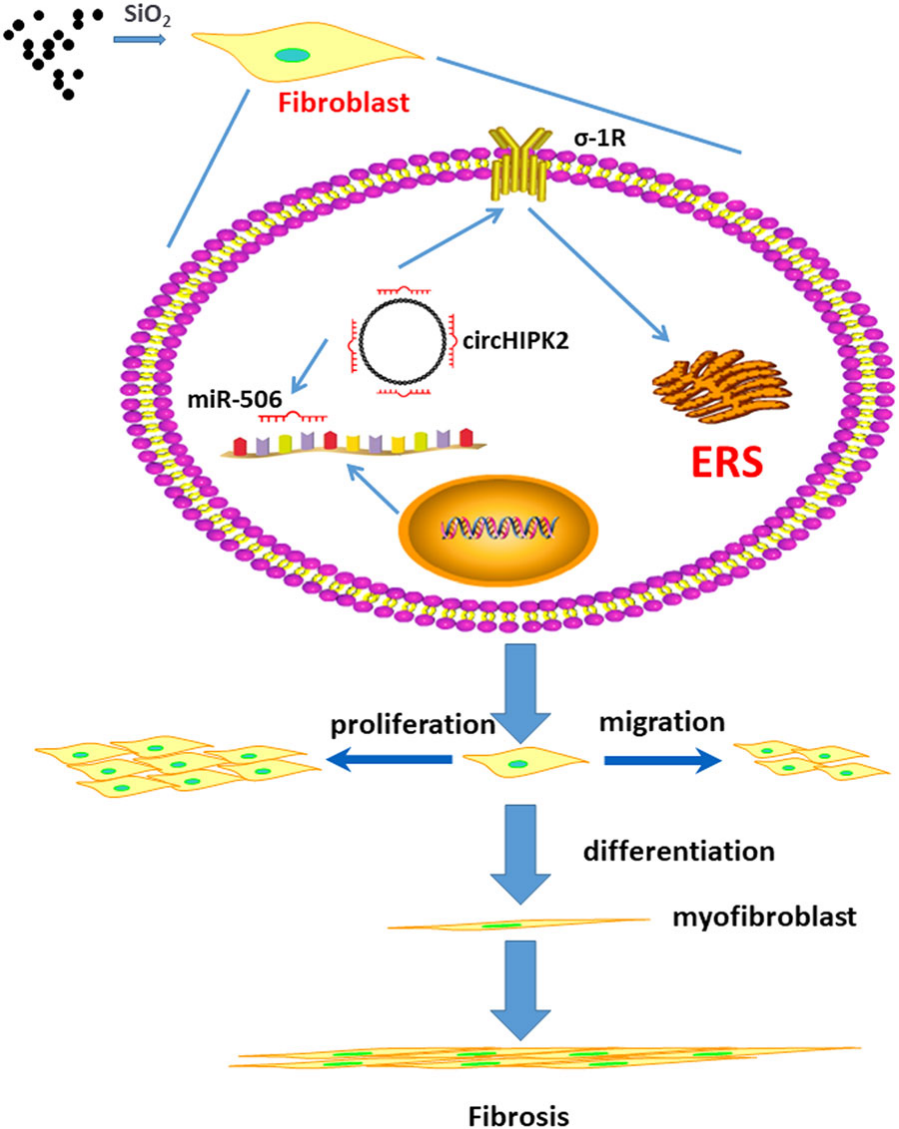
Schematic diagram showing the mechanisms by which circHIPK2/σ-1R in macrophages mediates silica-induced pulmonary fibrosis CircHIPK2 expression was increased in macrophages exposed to SiO_2_, leading to a subsequent increase in σ-1R expression. σ-1R promoted fibroblast activation. Fibroblasts showed enhanced proliferation and migration capacities and increased collagen synthesis, which may contribute to fibrosis induced by SiO_2_

Non-coding RNA is an important post-transcriptional mechanism to regulate proteins, whereas σ-1R regulation associated with non-coding RNA was recently reported^24^. Bioinformatic analysis identified circHIPK2, which has two complementary residues with miR-506-3p; a conserved miR-506-3p binding site within the σ-1R 3’-untranslated region was defined as the putative target of miR-506-3p (Fig. 6a), indicating that circHIPK2 may regulate σ-1R expression by acting as a ceRNA. As shown in Fig. 6b, FISH assays revealed the expression of circHIPK2 in HPF-a, in which, expression of circHIPK2 was upregulated after SiO_2_ exposure. Moreover, circHIPK2 and miR-506-3p levels were then detected by qRT-PCR (Fig. 6c–d). circHIPK2 showed a slight increase, whereas miR-506-3p remained stable with the time exposure of SiO_2_, indicating that ceRNA may be involved in σ-1R regulation. To further investigate the function of circHIPK2, siRNA was used to specifically silence the circRNAs (Fig. 6e). After silencing with circHIPK2-siRNA, σ-1R upregulation induced by SiO_2_ was attenuated (Fig. 6f–g). Moreover, circHIPK2-siRNA also inhibited the upregulation of BiP (Fig. 6h–i), collagen I and III (Fig. 6j–k) induced by SiO_2_, indicating that circHIPK2 was upstream of functional changes in HPF-a (Fig. 7)

.

## Discussion

Mounting evidence has suggested that pulmonary fibrosis was mainly caused by abnormal activation of alveolar macrophages and pulmonary fibroblasts (PFBs)^46^. As the straightforward impacts of SiO_2_ on PFBs tend to be relatively unnoticed, we focused on the effects of σ-1R expression in pulmonary fibroblasts on cell proliferation and migration after *in vitro* exposure to SiO_2_.

Several lines of evidence have demonstrated that σ-1R is a 25-kDa protein expressed in the endoplasmic reticulum that can facilitate cell proliferation and migration in the CNS. For instance, σ-1R receptor agonists exert potentiation of neurite outgrowth, myelination, long-term potentiation, and neuroprotection in *in vitro* studies and in animal models of neuropsychiatric diseases, such as ischemia and Alzheimer’s disease^47–53^. In the current study, SiO_2_-induced σ-1R upregulation promoted cell proliferation and migration in PFBs, indicating the general role of σ-1R in determining cell fate. Moreover, effect of SiO_2_ on Col I, Col III and σ-1R, as well as PERK and eIFα, was recovered after 24 h, which may due to the maximum effect reached. On the other hand, both ATF6α and BiP showed a rapid and sustained increase after SiO_2_ exposure because those two protein is upstream of other ERS marker, whereas further experiments need to confirm this different expression pattern of ERS marker.

The ER is an important organelle in the regulation of unfolded protein response (UPR), a protective mechanism accumulation of misfolded or unfolded proteins and autophagy, an intracellular degradation pathway for recycling and eliminating abnormal protein and damaged organelles^16–18,54^. These two mechanisms are correlative, confirmed by studies shed light on the ability of ERS to stimulate or inhibit autophagy. On the other side, the UPR is distinguished by the action of three signaling proteins: PERK, ATF6, and IRE1α^17^. Under physiological conditions, the luminal domains of PERK and ATF6 proteins are bound to the ER resident chaperone BiP, which keeps them inactivated^55^. It has been postulated that unfolded protein accumulation in the ER induces BiP release from these complexes to assist with the folding of accumulated proteins. Unlike these two associated with BiP, another UPR modulators IRE1α induces signal transduction events ameliorating misfolded protein accumulation in the ER by promoting the expression of ER chaperones, inhibiting protein entry into the ER via arresting mRNA translation and stimulating retrograde transportation of misfolded proteins from the ER into the cytosol for ubiquitination and destruction by a process known as ER-assisted degradation^55,56^. Therefore, in current study, SiO_2_ exposure to fibroblast increase the accumulation of misfolded protein, thus activating UPR, which leads to the upregulation of ERS related protein.

σ-1R is highly clustered at specialized ER subdomains that are physically associated with the mitochondrial outer membrane (MAM)^15^, a specialized ER membrane that regulates Ca^2+^ signal transduction, bioenergetics, cell death, and lipid metabolism^57,58^. At the MAM, σ-1R acts as a molecular chaperone and sustains the proper conformation of inositol triphosphate receptor type 3 to ensure proper Ca^2+^ signaling from the ER into mitochondria to facilitate ATP production^15,59,60^. The σ-1R also chaperones an ERS sensor, inositol-requiring enzyme 1, to ensure the proper transmission of ERS into the nucleus to call for the enhanced production of anti-stress and antioxidant proteins^61^. Previous study has elucidated that σ-1R is highly assembled in MAM, forming a complex with BiP under physiological conditions. Once ERS is activated, σ-1R dissociates from BiP and becomes an active molecular chaperone. Mounting evidence has demonstrated that σ-1R is involved in ERS activation in different settings. All these studies indicated that the mechanism of σ-1R was through ERS, whereas ERS was not thoroughly explored in silicosis. Here, we found that upregulation of σ-1R induced by SiO_2_ via ERS mediated a downstream cascade of fibroblast activation, indicating the therapeutic target of pulmonary fibrosis. Moreover, another of example of σ-1R involvement in disease development was unveiled, which is involved in cellular functional change and might be a general change in the disease pathology process.

Compared with studies on σ-1R function, research on its regulatory mechanism is far less. A recent study suggested a role for post-transcriptional mechanisms associated with non-coding RNAs in σ-1R regulation^24^. Newly found circRNAs form a class of novel non-coding RNAs that are relatively stable due to their cyclic structures. Therefore, circRNAs can resist excision enzymes, making them ideal disease marker candidates. circRNAs can act as molecular sponges to combine and block the function of miRNAs, namely, via the ceRNA mechanism. Bioinformatic analysis suggests that circHIPK2 and miR-506-3p may be involved in σ-1R regulation via ceRNA. Study indicates that long non-coding RNA NEAT1 facilitates pancreatic cancer progression through negative modulation of miR-506-3p^62^. CircHIPK2 is derived from exon 2 of the HIPK2 gene, acting as a sponge for several microRNA. A recent study reported that circHIPK2 can directly bound to miR-124 and acted as an endogenous sponge for miR-124 to inhibit its activity, thus activate astrocyte via the regulation of autophagy and ERS through σ-1R^27,62^. In addition, HIPK2 is reported to be a crucial driver of kidney fibrosis by regulating pro-apoptosis, pro-fibrotic and pro-inflammatory pethways^63^, which may be a potential target for antifibrotic. These evidences suggest that circHIPK2 has a significant role in cell fate determination. In current study found that circHIPK2 expression increased, whereas miR-506-3p remained stable, which matches the predicted ceRNA mechanism. Functional experiments and specific knockdown of circHIPK2 with siRNA confirmed the role of circHIPK2 in fibroblast activation induced by SiO_2_, suggesting that circHIPK2 may be a potential biomarker in diagnosing silicosis.

## Conclusions

Our study elucidated a link between SiO_2_-induced fibroblast activation and the circHIPK2/σ-1R pathway, thereby providing insight into the potential use of σ-1R to develop novel therapeutic strategies for treating silicosis.
